# The Panamanian health research system: a baseline analysis for the construction of a new phase

**DOI:** 10.1186/1478-4505-11-33

**Published:** 2013-09-04

**Authors:** Luz I Romero, Cristiane Quental

**Affiliations:** 1 Universidad Católica Santa María La Antigua, Dirección de Investigación, Apartado Postal 0819-08550, Panamá, República de Panamá; 2 Escola Nacional de Saúde Pública, Fundação Oswaldo Cruz, Programa de Doutorado em Saúde Pública, Rua Leopoldo Bulhões 1480, 21041-210, Rio de Janeiro, RJ, Brazil

**Keywords:** Health research policy, Health research system, Panama

## Abstract

**Background:**

In Panama, the health research system has been strengthened during recent years by the development of new financing opportunities, promotion of scientific and technological activities, and initiation of human capital training to ultimately improve competitiveness. However, aligning this system with the population’s health needs is a significant challenge. This study was designed to characterize the National Health Research System in Panama, aiming to understand it within a local context to facilitate policymaking.

**Methods:**

The study was based on the analysis of operative and functional components of the National Health Research System, characterized by four specific components: stewardship, financing, creation and maintenance of resources, and production and use of research results. The analysis was based on official documents from key local institutions in the areas of science, technology and innovation management, and health and health research, as well as bibliographic databases.

**Results:**

Panama’s National Health Research System is characterized by the presence of only two biomedical research institutes and reduced research activity in hospitals and universities, ambivalent governance, a low critical mass of researchers, reduced capacity to recruit new researchers, poor scientific production, and insufficient investment in science and technology.

**Conclusions:**

The present study illustrates an approach to the context of the Panamanian Health Research System which characterizes the system as insufficient to accomplish its operative role of generating knowledge for new health interventions and input for innovations. In turn, this analysis emphasizes the need to develop a National Health Research Policy, which should include longer-term plans and a strategy to overcome the asymmetries and gaps between the different actors and components of the current system.

## Background

At the country level, a health research system (HRS) is essential for producing and capturing health-related knowledge to improve the population’s health. The significance of these systems was once again recognized at the Latin American Conference on Research and Innovation for Health (Panama, 2011), where some of the central themes discussed included the importance of analyzing HRSs in order to strengthen these systems and optimize the concordance between knowledge management, public policies, research agendas, and health innovations [1].

In Panama, the mechanisms for managing the national science, technology, and innovation (ST&I) system have recently been remodeled, developing new financing opportunities, promoting scientific and technological activities, and initiating human capital training to ultimately improve competitiveness [2]. While these actions help to strengthen the Panamanian HRS, aligning this system’s efforts with the population’s health needs is a significant challenge. A recent study that analyzed the structure and function of 14 Latin American HRSs found heterogeneity and different degrees of development among the systems [3]. In the case of Panama, the study identified disarticulated structures, weak leadership, and the absence of an exclusive health research policy. In this regard, the lack of a contextual analysis is, perhaps, one of the most evident reasons why the decision-making process to advance the HRS’s functions remains difficult in Panama.

In the present study, Panama’s HRS was analyzed from a local perspective in order to gain an understanding of the system, identify weaknesses and facilitate decision making to establish a national health research policy.

### Conceptual framework

The international initiative to formulate a strategy that optimizes research production while improving health standards and promoting international collaboration gave origin to the concept of a HRS, defined as follows: “*the people*, *institutions*, *and activities whose primary purpose in relation to research is to generate high*-*quality knowledge that can be used to promote*, *restore*, *and*/*or maintain the health status of populations*” [4]. This definition is based on the integration of two concepts that allow for the structural elucidation and operational analysis of a HRS: the concept of a “system”, defined as “*a group of elements operating together to achieve a common goal*”, and the concept of health research, defined as “*the generation of new knowledge using the scientific method to identify and combat health problems*” [4]. The integration of these two concepts applies a systemic perspective that addresses the problem of research groups working in isolation and in areas of interest defined by the researchers themselves, by specific diseases or by institutional or market objectives [4].

In practice, applying the concept of HRS implies an, at times, unclear overlap of different systems, such as health, science and technology, education, and environment. In this respect, Sadana and Pang [5] specified the need to define the limits of health research topics, the institutions and individuals that participate in health research, and the average economic investment in this activity. With this in mind, four specific components were established to characterize HRSs: stewardship, financing, creation and maintenance of resources, and production and use of research results [5]. The greatest challenge of stewardship, the first component, is to create an efficient system with a high knowledge return from the investment dedicated to research [5]. This objective is guided by four operational factors: a) the national HRS’s definition; b) adherence to agenda priorities; c) establishment and monitoring of ethical research standards; and d) monitoring and evaluation of research [4]. Operationally, the availability of secure funds and accountable allocation of resources is the second component; meeting the HRS agenda objectives depends heavily on responsible financing. The third component, creation and maintenance of resources, consists of stimulating, maintaining and generating human resources and infrastructure to execute research. The fourth component is production and use of research results [5], which aims to share knowledge for innovation or use results as evidence for policymaking and new health interventions.

## Methods

The conceptual framework described above guided the characterization of the operational and functional components of Panama’s HRS in this descriptive study. For the contextual analysis of ST&I activities and financing, official reports from the National Secretariat of Science, Technology and Innovation (SENACYT) were used; the documents are available from the institution’s official web page [6]. The health situation was analyzed based on official documents from Panama’s Ministry of Health (MINSA), also published on MINSA’s web page [7]. To determine Panama’s scientific production, MEDLINE/PubMed and LILACS databases were searched using the key word “Panama” and publications from the year 2000 until December 2012 were included in the analysis. Only those publications with a principal author linked to a local, Panamanian institution were used. The number of researchers with a PhD degree at the Institute for Scientific Research and Technology Services (INDICASAT) in 2012 was obtained through personal communication with the institute’s Director.

## Results

### Context

Panama is a multiethnic country with a population of 3.7 million [8] and a human development index of 0.768, which is 58^th^ worldwide [9]. This is the highest index for Central America and the fifth among Latin American countries, surpassed only by Chile, Argentina, Uruguay, and Mexico (positions 44, 45, 47, and 48, respectively) [9]. Socioeconomically, Panama is distinct from other Central American countries due to the presence of the Panama Canal and its dollarized economy since 1904. Along with the intense commercial and financial activities derived from this interoceanic route, Panama has experienced continuous economic growth during the last decade. According to the World Bank [10], the service sector accounts for 75% of the country’s gross domestic product (GDP), which in 2011 stood at US$ 30.676 billion and US$ 12,200 per capita. The service sector has strongly contributed to establishing Panama as the second most globalized economy on the American continent (40^th^ place worldwide out of 144 analyzed countries in 2012), with only Chile ranking higher (33^rd^ place) [11]. However, structural problems persist in Panama, including poverty, marginalization, and inequality; 32.4% of the population lives below the poverty line and 14.2% of those live in extreme poverty, defined as less than US$ 1.77 per day per individual [12]. Indigenous groups account for 12.3% of the total population [8] and are the most affected by inequity; the extreme poverty rates range from 50% to 90% among these indigenous groups [12].

### Health situation

According to the 2012 WHO statistics [13], Panama invests 8.1% of its GDP in health, of which 68.1% is public and 31.9% is private sector investment. In comparison to the rest of Central America, Panama’s health investment is only surpassed by Costa Rica and Nicaragua (10.5% and 9.6% of GDP, respectively) and is below the median for all countries of the Americas, which is estimated at 14.4% of the GDP.

The health situation in Panama is one of a developing country going through a demographic transition, demonstrated by a low population growth rate (1.59%) and an increase in life expectancy at birth. During the past 10 years, life expectancy has varied from an average of 72.9 to 75.9 years for both sexes [14]. The mortality rate has varied from 4.7 to 3.4 deaths per 1,000 inhabitants over the past four decades [15]. At the same time, the population has experienced an epidemiological transition characterized by a decrease in mortality due to transmissible diseases and an increase in mortality and years of potential life lost due to non-transmissible diseases (mainly malignant tumors, ischemic heart disease, cardiovascular disease, and diabetes), accidents, and violence [16]. Disease caused by HIV/AIDS and pneumonia are the only transmissible diseases among the top ten causes of death in the country [15]. However, among the indigenous populations, the health situation is different; the principal causes of years of potential life lost are transmissible diseases (tuberculosis, HIV/AIDS, and diarrhea), followed by non-transmissible diseases (malnutrition, congenital malformations, chromosomal abnormalities, and diabetes) [16].

The Panamanian health system is composed of three distinct entities: MINSA, Social Security (CSS), and a private system. MINSA and CSS make up the public health system and cover 40% and 60% the Panamanian population, respectively, through a network of parallel services [17]. This system’s model of care is predominantly recuperative and directed to the most prevalent health problems with greater negative impact on the population, with less emphasis on preventive programs [18]. According to this scheme, there are specific functions attributed to each institution. MINSA, established in 1969 as the national health authority, is responsible for issuing public policies and addressing the spectrum of competencies and actions necessary to improve the population’s health and quality of life [19]. To meet this end, MINSA operates through five-year national plans that regulate and drive essential public health functions [14]. On the other hand, the assurance of health services, together with harmonizing the health system’s responsibilities with its expenses are functions that MINSA shares with the CSS, founded in 1941 [17]. The CSS provides health services and social security under the classical Bismarckian model characterized by assistance benefits, retirement, pensions, and subsidies based on employee salaries [18]. This dual health system results in an irregular distribution of health networks, with a greater concentration of resources and spectrum of services available in urban areas and less in remote and/or rural regions. As a result, the system remains fragmented, leading to exclusion of marginal populations from access to basic health services and, consequently, internal inequalities in the country [18]. The private health system provides additional coverage for 15% of the population through a series of medical and hospital service plans provided by a network of private institutions, including 12 medical centers and a number of clinics located predominantly in the metropolitan region. The income generated by the private health sector represents 0.9% of the national GDP; this sector is expanding with a 3% annual growth [20].

### Description of the HRS

Panama does not have a research and innovation policy specific to health [3], instead there are a series of ST&I and health policies which address these issues together (Table 1). The Health Code of 1947 [21], which is still in effect, was the original regulation related to public health and hygiene, health policy, and preventive and curative medicine. Within the Code, scientific research is recognized as a necessary resource and guide to address health problems. The transfer of the Gorgas Memorial Laboratory, a research institution working on tropical diseases, which had been run by the United States since 1921, to MINSA in 1990 represented an important initiative to link research with the National Health System. Since 1997, along with the change in the institution’s name to Gorgas Commemorative Institute of Health Studies (ICGES), it has been recognized as the institution in charge of national research stewardship [22]. Subsequently, Law 78 of 2003 restructured ICGES as a public entity with legal, financial and technical autonomy, responsible for driving national scientific research in the area of health, in cooperation with MINSA [23]. Recently, through executive decree No. 1 of January 21, 2013, the National Committee on Ethics for Research in Panama was established within MINSA, with the objective to promote good clinical practices for human subjects’ protection in the country [24]. Furthermore, in Policy 7 of the current National Health Policy and Strategic Plan 2010–2015 [14], a series of guidelines and action plans were established focused on strengthening research as a means to improve public health, but these plans have not been implemented.

**Table 1:** Legal framework for the National Health Research System in Panama

**Health-related laws/policies**	**Established outcome**
Law 66 of November 10, 1947 [21]	Code that regulates health issues and recognizes the importance of research to resolve health problems.
Law 78 of December 17, 2003 [22]	Grants autonomy to ICGES and responsibility for conducting, strengthening and developing scientific health research in the country.
Executive decree 1 of January 21, 2013 [24]	Creation of the National Committee on Ethics for Research within MINSA to promote good clinical practices for human subjects’ protection in the country.
National Health Policy 2010–2015 policy 7 [14]	Institutional strengthening of MINSA in health research to improve public health at a national level.
**ST&I-related laws/policies**	
Law 13 of 1997 [2]	Gave SENACYT autonomy for national development of ST&I activities.
Law 50 of 2005 [2]	Gave SENACYT jurisdiction over policies and resources for ST&I in Panama.
Law 56 of December 14, 2007 [25]	Formation of the National Research System to recognize excellence in human resources and institutions according to a merit system.
National Strategic Plan on ST&I 2010–2014, [2]	Program for development in ST&I in biosciences and health sciences, and an agenda with strategic actions in research, development and innovation for health.

On the other hand, SENACYT has independently been the key actor in opening and promoting research activities, innovation and human resource development, including work within the health sector. The Secretariat was established by a decree in 1992 as a joint organization of the Panamanian Presidency, but later legislation gave SENACYT autonomy (Law 13 of 1997) and jurisdiction over policies and resources for science and technology (S&T) in Panama (Law 50 of 2005) [2]. In 2007, SENACYT created the National Research System (NRS) through Law 56; the objective of this system is to stimulate the country’s scientific productivity in all knowledge areas and to recognize excellence in institutions and individual researchers through a peer-reviewed merit system [25]. SENACYT is also responsible for coordinating the strategic plan for ST&I (PENCYT), from which the financing to execute programs outlined in the national agenda of this five-year plan is derived. The ‘Program for scientific and technological development and innovation in biosciences and health sciences’ is one of the seven sector plans that makes up PENCYT 2010–2014, together with plans for agriculture, industry and energy, logistics and transportation, basic sciences, social sciences, and education [2]. *Ad hoc*, sector-based commissions, formed by researchers, academics and managers brought together by SENACYT, construct these plans. Along with each plan, the priority agendas are developed. However, PENCYT’s biosciences and health sciences agenda is only an approximation of a national health research agenda, as it is not exclusive to health science; adopted by MINSA (the national health authority) nor has it been fully promoted by ICGES (the institution in charge of national research stewardship) at the national level.

### Investment in research

Recent data indicate that Panama invests 0.26% of GDP in ST&I [2], which is lower than the average for Latin America and the Caribbean at 0.7% [26]. Since 2005, SENACYT has implemented concrete policies and actions for financial support in the form of scholarships, researcher incentives, scientific infrastructure improvements, support for research and development projects in research centers and international collaborations for training and re-insertion of scientific talent, totaling an approximate investment of US$ 30 million per year Additional file 1 (Table 2). In coordination with the Institute for the Formation and Development of Human Resources (IFARHU), a state agency responsible for disbursements, a total of 896 scholarships [27] with an investment of 18 million dollars [28] have been awarded for students to study in areas where Panama lacks academic programs of excellence. Of the scholarship recipients, 37% have concluded their programs and 63% are still actively pursuing studies in 23 countries throughout the Americas and Europe. A total of 27.6% of the scholarships correspond to doctorates, 28% are for master’s degrees, 15% are for undergraduate studies in the area of education, 28.4% are for other areas, and only 0.9% corresponds to postdoctoral studies. Of the 568 active scholarships, 184 are doctoral, of which most are in the area of biology (29%), followed by engineering (19%), health (12.5%), and information technology (11%) [27] (Table 3).

**Table 2:** **Cumulative public funds invested in ST**&**I activities through SENACYT**, **2006 to 2012**

**Indicator**	**Value**
Panamanian investment in ST&I according to GDP [2]	0.26%
**SENACYT’s investment in ST&I activities for all areas in 2012 Additional file **1**:**	
- Investments in R&D projects, all areas	$6,789,000.00
- Innovation and competitiveness	$8,794,500.00
- Researcher incentives	$3,082,400.00
- Technological development	$4,050,000.00
- Training and technological transformation	$4,680,000.00
- Metrology and standards	$1,704,100.00
- Equipment	$800,000.00
**Total**	**$30,000,000.00**
**Masters, doctoral and postdoctoral scholarships for all areas, 2010 **[28]	**$18,000,000.00**
**Accumulated investment in R&D projects in all areas, 2006 to 2011 **[29]**:**	
- Environment and ecology	$4,587,942.00
- Biomedicine (health)	$4,351,099.00
- Engineering and IT	$2,809,049.06
- Agriculture	$2,587,598.00
- Infrastructure	$1,868,538.00
- Social sciences	$1,468,506.00
- Hard sciences	$837,321.00
- Logistics and transportation	$498,173.00
**Total**	**$19,008,226.06**

Monetary values are reported in US$.

Source: Data provided by SENACYT (senacyt.gob.pa).

**Table 3:** **Scholarships granted by SENACYT between 2006 and 2012** (**Total 896**)

**Degree**	**Completed scholarships**		**Active scholarships**		**Total scholarships**	
	**All sectors**	**Health sector only**	**All sectors**	**Health sector only**	**All sectors**	**Health sector only**
Postdoctoral	4	1	4	1	8	2, 25%
Doctoral	64	18	184	23	248	41, 16.5%
Master’s	93	6	158	15	251	21, 8.4%
Graduate	105	0	30	0	135	0, 0%
Under- graduate	62	0	193	0	255	0, 0%
Total	328, 37%		568, 63%		896	64, 7.2%

The number of scholarships for all sectors and the health sector only are shown. The number of health sector only scholarships is also shown as a percentage of all the scholarships awarded. The numbers of completed and active scholarships are shown as percentages of the total scholarships awarded. Source: Data provided by SENACYT [27].

According to official data, between 2004 and 2012, 339 research and development (R&D) projects totaling US$ 19,008,226.06 were financed by public funds given through project grants from SENACYT [29]. Of this funding, 24.2% was awarded to finance 85 ecology and environment research projects, representing 25% of the total financed grants (Table 4). The second largest group of grants awarded corresponded to 63 health science projects, accounting for 18.6% of the total financed grants and 22.9% of the total R&D investments (US$ 4,351,099). Interestingly, 89.2% of the funds designated for projects in biosciences are concentrated in three institutions: ICGES, INDICASAT, and Universidad de Panama (UP) (US$ 1,552,148, US$ 1,735,397, and US$ 593,739, respectively). On the other hand, 10.8% of the remaining projects are distributed among only five universities and hospitals (Table 5). Recent data indicate a similar amount of external funds are provided by international companies and agencies for biomedical research in Panama [30].

**Table 4:** **Distribution of public funds for 339 R**&**D projects from 2004 to 2011**

**Area/sector**	**n of projects**	**% of total number of projects**	% of investment*
Environment/ecology	85	25.0	24.1
Health sciences	63	18.6	22.9
Engineering and IT**	59	17.4	14.8
Agriculture	52	15.3	13.6
Social sciences	34	10.0	7.7
Hard sciences	18	5.3	4.4
Infrastructure	15	4.4	9.8
Logistics and transportation	13	3.8	2.6
**Total**	**339**	**100**	**100**

*Percentage of investment in all projects ($19,008,226.06).

**Information technologies.

Source: Data provided by SENACYT [29].

**Table 5:** Health science projects financed by SENACYT 2004–2011

**Institution**	**n of projects**	**% of projects**	**Investment (US$)**	**% of investment**^**a**^
ICGES	25	39.7	1,552,148.00	35.6
INDICASAT-AIP	21	33.3	1,735,397.00	39.8
Universidad de Panamá	9	14.3	593,739.00	13.6
Universidad Autónoma de Chiriquí	3	4.8	244,751.00	5.6
Caja del Seguro Social (CSS)	2	3.1	129,672.00	3.0
Hospital del Niño	1	1.6	15,792.00	0.4
Universidad Latina	1	1.6	40,000.00	1.0
Universidad de las Américas	1	1.6	39,600.00	1.0
**Total**	**63**	**100**	**4,351,099.00**	**100**

^a^Percentage of investment in health and bioscience projects only.

Source: Data provided by SENACYT [29].

### Scientific production

Recently, Barreto et al. [31] analyzed the publication production on epidemiology by 41 countries in the Latin American and Caribbean region; after Belize (6.8%), Panama was the country that showed the lowest annual growth (11.7%) over the past five decades (1961–2010). Panama was also designated as the country with the least number of publications per million inhabitants among all the countries analyzed, with an average of 5.21 publications in 2010, representing a 57% decrease in comparison to 1990 (9.12 publications per million inhabitants). However, an increase in local scientific production has recently been observed; a total of 90 publications, including biomedical and epidemiological studies, were produced by Panamanian institutions in the period from 2010 to the end of the present study (December 2012). Of these, 36 research articles were published by ICGES, equivalent to 83.7% of the institution’s production over the previous decade (n = 43). INDICASAT reported 31 publications for this same period, representing a 239% increase in production compared to the previous decade (n = 13). In addition, UP reported nine publications, while local hospitals and other institutions produced ten and four publications, respectively.

### Human resources and institutions

According to recent figures, Panama only has 476 full-time equivalent researchers in all sectors, with a ratio of 0.3 per 1,000 members of the economically active population [2]. These values are low in the context of Latin America, where the average number of researchers is 1.0 per 1,000 members of the economically active population [30]. The index is even lower when looking specifically at biomedical researchers. According to a 2008 Survey of Specialized Indicators of Biomedicine conducted by SENACYT, a total of 128 researchers were reported [30]. This number corresponded to 0.08 researchers per 1,000 members of the economically active population, of which only 79% (n = 101) were full-time equivalent. Of the 128 researchers, 28.9% were from ICGES (n = 37), 17% were from UP (n = 22), and 5.5% were from CSS (n = 7), while INDICASAT, Universidad Tecnológica, Hospital del Niño, Hospital San Miguel Arcángel, and the Panama Institute of Agricultural Research each represented 4.7% of researchers (n = 6 for each institution). The remaining 18 institutions had between one and five researchers each [30]. Most of the researchers reported having a master’s degree (55%) and 18% reported having an undergraduate degree only. A smaller percentage corresponded to doctoral (14%) and postdoctoral researchers (5%). However, the number of staff and associates with a doctoral degree at INDICASAT has increased significantly from 6 in 2008 to 25 in 2012.

Looking closer at the history of health research in Panama, the ICGES has a tradition of nearly nine decades of studies in the areas of emerging and zoonotic diseases, medical entomology, parasitology, virology and biomedical studies [32]. ICGES has been under Panamanian administration for only the past two decades, and this institution is currently responsible for providing health reference services at both the national and international level; since 1999, ICGES has served as the Central Public Health Reference Laboratory. In addition, by law, ICGES is responsible for managing and stimulating the HRS in coordination with MINSA [22]. ICGES has a three-year Strategic Operative Plan that was established in 2012. The Plan describes objectives and activities to strengthen research capacity at the national level through public health policies, to establish research priorities and to stimulate diffusion of health research and the formation of strategic alliances [32].

Along with the return of the Panama Canal Zone from the United States to Panama, the City of Knowledge was established in 1995 in order to transform the 120 hectares and infrastructures of the Clayton military base into facilities to promote sustainable development based on knowledge. Local and international academic and research institutions have been congregated within the City of Knowledge to promote scientific, technological, and innovation activities. Among these institutions, INDICASAT was established in 2002 through the support of SENACYT to increase Panama’s productive capacity while filling critical gaps in the scientific sector [33]. With these objectives, and through a rapid reorganization process, INDICASAT has been consolidated into a biomedical and environmental research institute. This has opened new niches for basic and applied research in the areas of neuroscience, molecular and cell biology, and drug discovery, as well as local biodiversity and clinical, epidemiological and translational research [33]. The rise in doctoral researchers at INDICASAT has also translated into an increase in the institute’s scientific productivity as indicated above. At the same time, INDICASAT is contributing to human capital training by establishing Panama’s first doctoral program in biotechnology, with support from Acharya Nagarjuna University in India. The program is projected to produce 14 doctoral graduates per year beginning in 2016 [33].

Meanwhile, as an academic institution, UP has a concentrated infrastructure for teaching and research in basic sciences and a network of laboratories; a few research groups have maintained low but sustained scientific productivity in the areas of pharmacology, parasitology, chemistry, and molecular biology. The number of biomedical researchers (n = 31) at UP has not varied significantly since 2008; 14 of these researchers have a master’s degree, while 8 have doctorates [29]. For institutional reasons, these researchers often have academic responsibilities which outweigh their commitment to conducting research and thus, limit scientific production. The university, in addition to undergraduate programs in health sciences (including medicine, dentistry, nursing, medical technology, and pharmacy), offers a series of master’s programs in basic sciences, clinical sciences and public health, which graduate an average of 40 students per year, but UP does not have any academic doctoral programs.

Despite the fact that Panama has 61 hospitals and 850 health facilities [14], the lack of a medical culture focused on research and the lack of researchers within hospitals limits research practice in these institutions. However, hospitals have also maintained low but sustained research activity in clinical treatment evaluation, therapeutic procedures, and vaccine development. Additionally, epidemiologic studies and clinical case studies are carried out through the individual efforts of a few medical doctors, who reported a total of six publications in 2011.

Proportionally, the distribution of researchers in the NRS reflects the institutions analyzed above; 32 (30.7%) of the 104 NRS researchers work within the area of health, of which 8 work at ICGES, 10 are at INDICASAT, 7 are in hospitals, 4 are at UP, and 3 are at other institutions [34].

### Research impact

In accordance with the previously mentioned study [30], Panama has a low impact in the area of biomedical research when considering the number of patents produced by the country, the application of products generated by research, the development of projects and employment generation. This study indicates that 6 of the 8 patents reported between 2002 and 2008 corresponded to intellectual property patents for new bioactive molecules generated by one local collaboration project (INDICASAT and UP) and one international collaboration (Smithsonian Tropical Research Institute (STRI) and Oregon State University). ICGES has achieved research impacts through public health interventions for transmissible diseases, tropical diseases and vector control, among others [32].

## Discussion

Only two biomedical research institutions in the entire country, reduced health research activity in hospitals and universities, a low critical mass of researchers, poor national scientific production, and insufficient investment in ST&I activities are the major characteristics of Panama’s current national HRS. Nonetheless, this situation may evolve, depending on the continuity and strengthening of national investment plans for science and health research.

The rates at which different Latin American countries have developed systems of research and innovation in health have been extremely unequal. Countries like Brazil, Mexico, and Chile have consolidated their systems, creating resource and process structures for knowledge management which is then incorporated into national productivity through health innovations [3]. In the case of Panama, the current panorama shows low scientific production indicators contrasted with high indices of economic development. The present analysis demonstrates how the local national HRS, until a decade ago, was sustained by a single health research institution, ICGES. A lower but sustained role has been played by the UP. Other components of the system (stewardship, financing, creation and maintenance of resources, and production and use of research results) were sustained from the outside until the 1990s during the United States’ administration of Gorgas Memorial Laboratory [31]. While SENACYT is now recognizing the need for sustainability and creating resources, this highlights the governance insufficiencies of MINSA, which is essential to conducting and coordinating health research throughout the country. A recent study that evaluated the national HRSs of 14 Latin American countries [3] reported that Brazil and Costa Rica have an administration led by their ministries of health, while the majority of the countries analyzed have mixed administrative structures, such as a ministry of health in addition to a S&T agency. In the study by Alger et al. [3] a disarticulation in Panama’s HRS was identified, as the governance role was given by law to a research institution with financial and technical autonomy from MINSA. In practice, this has diffuse effects, being that stewardship is charged with establishing the system objectives and management structure for planning and executing action plans. Through the stewardship function, the leading institution is also responsible for coordinating collective participation among actors that make up the system, including those from the State, academia, and private or civil society. However, in the Panamanian HRS model, the national health research plans are set by SENACYT through the health research agenda established in the PENCYT. Although these plans have been designed by inter-institutional collaboration formed by SENACYT, the lack of a national research agenda dictated by the central leadership in health (MINSA) may limit the alignment of health needs with the agenda’s lines of actions, as has occurred in Panama. Therefore, in order to improve the local HRS and its articulation, the stewardship subject deserves a broader discussion including the revision of related aspects within law 78. Likewise, MINSA is currently in discussion regarding the issues of sustainability and efficiency within their own system, while also considering instituting a unified health system for Panama. However, discussion on establishing an exclusive health research policy, including an agenda of priorities assumed by the country’s health leadership, is also necessary and deserves to be discussed.

Contributing to the difficulties in strengthening Panama’s national HRS through human capital is the lack of doctoral programs in the country. The need to train doctoral students in other countries and the recent opening of the only biomedical doctoral program in Panama with the support of Acharya Nagarjuna University, India, emphasize this fact. UP, the main academic entity in the country, has tried to overcome internal difficulties to improve academic programs, creating a series of master’s programs in basic sciences, clinical sciences and public health that together produce an average of 40 students per year. Another aspect that needs to be addressed is the ratio of outside doctorates for the area of biology *vs*. health, which is equivalent to 2.3:1. This ratio is not surprising being that there is an important tradition of biology and ecology research in Panama, first led by the Gorgas Memorial Laboratory and currently influenced by the STRI. This institute is a United States government entity that has been present in Panama since the beginning of the 20^th^ century and was associated with the Canal’s construction; its mission is to study tropical biology [35]. This institute receives federal financing from the United States, has a highly qualified staff and among the best land and marine infrastructure for basic research in Panama, as well as high scientific productivity. However, STRI is mistakenly accounted for as a national resource, which influences Panama’s ST&I indicators. For the purposes of the present study, STRI was not included because it is neither a health research institution nor a national entity.

The national HRS’s reduced capacity to recruit new researchers with doctoral and postdoctoral training, sponsored by SENACYT–IFARHU’s initiatives to increase the human capital in the country, is a constant concern. The national HRS’s current structure based on two biomedical research institutions with scarce, small research groups and the limited R&D activity in academic institutions and hospitals without a research culture do not provide adequate space for inserting a new researcher labor force. This situation can only be overcome by significant political will to strengthen the HRS within national development plans with a vision that values knowledge as important evidence for decision making for health interventions and encouraging innovation. Among the next steps, construction of the Panama Research Institute of Science and Medicine has started; this will be a 25,000 m^2^ complex of infrastructures located within City of Knowledge. With over $25 million in investments, this complex will increase laboratory space for INDICASAT and for the National Metrology Center, as well as the SENACYT’s new headquarters [2]. Together, these actions are projected to increase Panama’s capacity for biomedical research and recruitment of new researchers. Simultaneously, CSS has initiated construction of the Hospital City of Panama, a 31.9-hectare former military area, with a $587 million investment. The principal objective of this construction is to establish a complex of new hospital infrastructure to modernize health services for social security beneficiaries who represent more than 60% of the population. In addition to the hospital complexes, the new infrastructure will house ICGES and UP’s School of Medicine, presenting a unique opportunity for inter-institutional alignment to promote biomedical, clinical, and public health research. Panama is also expecting a significant increase in its GDP beginning in 2014, following the Canal expansion after a $5.25 billion investment [36].

## Conclusions

The present study illustrates an approach to the context of the Panamanian HRS which characterizes the system as insufficient to respond to the health needs of the population, despite recent efforts to strengthen such system. In turn, this analysis emphasizes the need to develop a National Health Research Policy in Panama, which should include longer-term plans. Such a policy could serve as an element to integrate national health planning and the population’s sanitary needs. Under this vision, the asymmetries and gaps between the different actors and components of the current system could be overcome, ultimately transforming Panama’s HRS into an operative instrument that generates knowledge to be used as evidence for new health interventions and input for innovations.

## Abbreviations

CSS: Social Security; HRS: Health Research System; ICGES: Gorgas Commemorative Institute of Health Studies; IFARHU: Institute for the Formation and Development of Human Resources; INDICASAT: Institute for Scientific Research and Technology Services; MINSA: Panama’s Ministry of Health; NRS: National Research System; PENCYT: Strategic plan for ST&I; SENACYT: National Secretariat of Science Technology and Innovation; ST&I: Science technology and innovation; STRI: Smithsonian Tropical Research Institute; UP: Universidad de Panama.

## Competing interests

The authors declare that they have no competing interests.

## Authors’ contributions

LR was responsible for the concept of the study, collected and analyzed the relevant data, and drafted the manuscript. CQ contributed to the overall concept of the study, provided guidance on the paper’s intellectual content, and refined the original version of the manuscript. Both authors read and approved the final manuscript.

## Authors’ information

LR is currently the Research Director of Universidad Santa Maria la Antigua at Panama. Previously, the author served as staff scientist and Acting Director of INDICASAT as well as being a member of the executive directors committee at SENACYT during the establishment of the national science and technology operative management mechanisms. Currently, LR is also a PhD graduate student in public health, within the sub-area of science and technology, at the National School of Public Health, FIOCRUZ, Rio de Janeiro, Brazil. CQ is currently a Professor in the Department of Planning and Analysis, at the National School of Public Health, FIOCRUZ, Rio de Janeiro, Brazil. CQ’s expertise is in the field of ST&I analysis, and this author mentored LR in the current study as a component of her doctoral thesis on public health.

## Supplementary Material

Additional file 1 **Public investment for year 2012 on ST&I activities for all areas of knowledge through SENACYT.** The official data was accessed on SENACYT’s web page (senacyt.gob.pa) on August 2, 2012. Click here for file
