# Supplementary material for: The Panamanian health research system: a baseline analysis for the construction of a new phase
Source: Health Res Policy Syst. 2013 Sep 4;11:33. doi: 10.1186/1478-4505-11-33 (PMC3847496; doi:10.1186/1478-4505-11-33)
Supplement: Additional file 1 — Public investment for year 2012 on ST&I activities for all areas of knowledge through SENACYT. The official data was accessed on SENACYT’s web page (senacyt.gob.pa) on August 2, 2012. [file 1478-4505-11-33-S1.pdf]

SECRETARIA NACIONAL DE CIENCIA, TECNOLOGIA E INNOVACION

Direccion de Administración y Finanzas

Departamento de Presupuesto

Ejecución Presupuestaria de Inversiones

(En balboas)

Partida	Programas y Proyectos de Inversión	Presupuesto Ley Modificado 1	Asignado a la Fecha 2	Ejecutado a la Fecha 3	% de Ejecución 4 = (3/2 * 100)	Pagado 5	Saldo a la Fecha 6 = 2-3
1.32.1	**TOTAL**	**30,000,000**	**2,045,180**	**418,144**	**20.4**	**397,069**	**1,627,036**
	**Investigación Científica**	**6,789,000**	**194,287**	**30,811**	**15.9**	**30,442**	**163,476**
1.32.1.1.703.01.06	INDICASAT	2,000,000	0	0	0.0	0	0
1.32.1.1.703.01.09	Apoyo y Promo. de Actividades de Inv.	4,789,000	194,287	30,811	15.9	30,442	163,476
	**Obras, Remodelaciones y Equipa.**	**800,000**	**63,165**	**13,044**	**20.7**	**13,044**	**50,121**
1.32.1.1.703.02.12	Obras, remodelaciones y Equipo	800,000	63,165	13,044	20.7	13,044	50,121
	**Metrología y Normas**	**1,704,100**	**304,100**	**0**	**0.0**	**0**	**304,100**
1.32.1.1.703.03.01	Metrología y Normas	1,704,100	304,100	0	0.0	0	304,100
	**Innovación y Competitividad**	**8,794,500**	**551,074**	**56,841**	**10.3**	**53,680**	**494,233**
1.32.1.1.703.04.10	Innovación Empresarial	2,550,000	239,459	15,561	6.5	15,561	223,898
1.32.1.1.703.04.11	Fortalecimiento del Sistema Nacional de Ci	5,403,400	276,442	41,280	14.9	38,119	235,162
1.32.1.1.703.04.13	Desarrollo del Plan Estrátegico de C y T	841,100	35,173	0	0.0	0	35,173
	**Estímulos e Investigaciones**	**3,082,400**	**175,501**	**44,546**	**25.4**	**43,240**	**130,955**
1.32.1.1.703.06.01	En el aprendizaje de las ciencias	3,082,400	175,501	44,546	25.4	43,240	130,955
	**Desarrollo Tecnológico**	**4,050,000**	**314,212**	**33,167**	**10.6**	**16,928**	**281,045**
1.32.1.1.703.09.02	Programa de Infoplazas	1,760,000	132,785	4,680	3.5	4,200	128,105
1.32.1.1.703.09.26	Nac. Indicadores de Ciencia y Tec	240,000	20,283	6,851	33.8	6,851	13,432
1.32.1.1.703.09.27	Clusters de competitividad	1,450,000	114,000	14,742	12.9	0	99,258
1.32.1.1.703.09.28	Mejoramiento Automatización Senacyt	600,000	47,144	6,894	14.6	5,877	40,250
	**Capacitación**	**250,000**	**21,113**	**8,490**	**40.2**	**8,490**	**12,623**
1.32.1.1.703.13.03	Recurso Humano carrera administrativa	250,000	21,113	8,490	40.2	8,490	12,623
	**Transformación Tecnológica**	**4,530,000**	**421,728**	**231,245**	**54.8**	**231,245**	**190,483**
1.32.1.1.331.14.01	1987/OC-PN Transformacion tecnologica	2,700,000	266,650	197,080	73.9	197,080	69,570
1.32.1.1.703.14.01	1987/OC-PN Transformacion tecnologica	1,830,000	155,078	34,165	22.0	34,165	120,913

Fuente: Reporte del SIAFPA
